# Diseases of the Nervous System

**Published:** 1898-12

**Authors:** 


**Diseases of the Nervous System.** By CHARLES E. BEEVOR, M.D. Pp. xvi., 432. London: H. K. Lewis. 1898.

The subject of this excellent book is such a large one, that it is a matter of no ordinary difficulty to write a volume of moderate size without omitting anything really essential. At the same time, students and general practitioners need a book to which they can readily refer, and which will enable them to lay a good groundwork of knowledge of nervous diseases which they can afterwards supplement. The author has performed his task very well, and the amount of information that he has managed to afford his readers and yet keep his book of small dimensions is remarkable. At the same time, the book is not a mere abstract or compilation, but is evidently the work of one who has had a large practical experience of his subject and is speaking out of the fulness of his own knowledge. The descriptions are everywhere clear and the style readable; brevity in this case has not, as it so often does, led to obscurity in style.

There is a short anatomical introduction, which gives the salient facts of the anatomy and physiology of the nervous system, and then follow two useful chapters on the methods of case-taking and the modes of examination of patients. The rest of the book is taken up with the systematic description of the various diseases of the brain, cord, and nerves, considered in each case under the heads of causes, symptoms, pathology, diagnosis, prognosis, and treatment.

We think that this book will be found a very useful one, and that the reader may turn to it for trustworthy and accurate information and a scientific grounding in the knowledge of nervous disease: not the least valuable point is its essentially practical character.

